# Characterization of the complete plastid genome of *Rauvolfia verticillata* (Apocynaceae), with its phylogenetic analysis

**DOI:** 10.1080/23802359.2019.1693287

**Published:** 2019-11-21

**Authors:** Wenna Chen, Weiling Liang, An Li, Jie Ma

**Affiliations:** College of Landscape and Ecological Engineering, Hebei University of Engineering, Handan, China

**Keywords:** *Rauvolfia verticillata*, Apocynaceae, plastid genome, phylogenomics

## Abstract

*Rauvolfia verticillata* is a medical plant (Apocynaceae) widely distributed from India to China, the Indo-China Peninsula, Indonesia, and the Philippines. The first complete plastid genome sequence of the species reported here was 155,856 bp in length, with the large single-copy (LSC) region of 86,085 bp, the small single-copy (SSC) region of 18,299 bp, and two inverted repeats (IRa and IRb) of 25,736 bp. The plastome contained 113 unique genes, including 79 protein-coding genes, 4 ribosomal RNA genes, and 30 transfer RNA genes. The overall GC content was 37.92%. The result from phylogenetic analysis suggests that *Rauvolfia* is closely related to the genus *Catharanthus*.

The genus *Rauvolfia* L. in the family Apocynaceae includes about 60 trees or shrubs species widely distributed in Africa, Asia, and America (Li et al. 1995; Endress et al. 2014), with about seven species in China (Li et al. 1995). *Rauvolfia verticillata* (Lour.) Baill. is a species of shrubs widely distributed in India, Sri Lanka to China, the Indo-China Peninsula, Indonesia, and the Philippines (Li et al. 1995). In China, the species is used medically to treat snake poisoning, malaria, and typhus (Li et al. 1995, 2015; Chen et al. 2019).

The fresh leaves of *R. verticillata* were collected from Guangzhou (Guangdong province, China; Coordinates: N23°11′20″, E113°21′53″). Voucher specimen (Chen et al. 2019) was deposited in the Herbarium of South China Botanical Garden, Chinese Academy of Sciences (IBSC). Total DNA was isolated from fresh leaves following the modified CTAB method (Doyle and Doyle 1987). Whole genome was sequenced on the Illumina HisSeq 2500 Sequencing System. The filtered reads were assembled using the program NOVOPlasty (Dierckxsens et al. 2017) with the complete plastid genome of *Catharanthus roseus* (NC_021423) as reference. The genome obtained was annotated using software PGA (Qu et al. 2019). The annotated plastid genome sequence has been deposited into the GenBank with the accession number MN480804.

The plastome of *R. verticillata* with 37.93% GC content is 155,856 bp in length. Structural analysis of the complete plastid genome exhibits a typical quadripartite circular structure. It contained a large single-copy (LSC) region of 86,085 bp and a small single-copy (SSC) region of 18,299 bp, which were separated by two inverted repeat regions (*IRa* and *IRb*) of 25,736 bp. A total of 113 unique genes were annotated, including 79 protein-coding genes, four ribosomal RNA genes (rrn16, rrn23, rrn4.5, and rrn5), and 30 transfer RNA genes. In the two IR regions, 17 gene genes were duplicated including six protein-coding genes (*ndhB*, *rpl2*, *rpl23*, *rps12*, *rps7*, and *ycf2*), four ribosomal RNA genes (rrn16, rrn23, rrn4.5, and rrn5) and seven transfer RNA genes (trnA-UGC, trnI-CAU, trnI-GAU, trnL-CAA, trnN-GUU, trnR-ACG, and trnV-GAC).

To investigate the phylogenetic position of *R. verticillata*, the plastid genome of the species obtained in the present study and other twenty published plastid genomes of the family Apocynaceae were used to construct a phylogenetic tree using *Gelsemium sempervirens* (MG963263) and *Gentiana officinalis* (NC_039574) as the outgroups. The result from the maximum likelihood (ML) phylogenetic analysis based on 79 protein-coding genes of 21 representative species within the family Apocynaceae suggests that *R. verticillata* is closely related to the genus *Catharanthus* G. Don (Figure 1).

**Figure 1.**
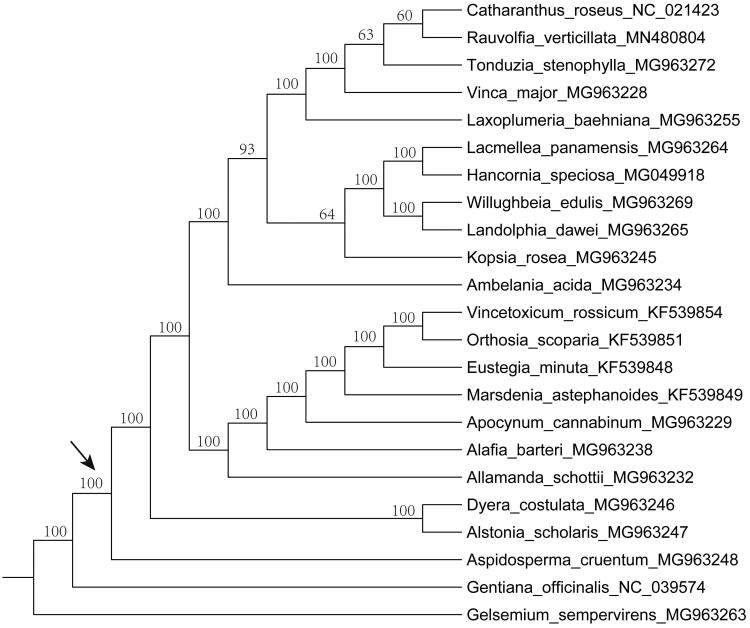
Maximum likelihood tree inferred from 79 protein-coding genes of 23 plastomes. Bootstrap values are indicated above branches. The crown node of Apocynaceae is shown by an arrowhead.
